# Gold-catalyzed heterocyclizations in alkynyl- and allenyl-β-lactams

**DOI:** 10.3762/bjoc.7.73

**Published:** 2011-05-17

**Authors:** Benito Alcaide, Pedro Almendros

**Affiliations:** 1 Grupo de Lactamas y Heterociclos Bioactivos, Departamento de Química Orgánica I, Unidad Asociada al CSIC, Facultad de Química, Universidad Complutense de Madrid, 28040-Madrid, Spain; 2 Instituto de Química Orgánica General (IQOG), Consejo Superior de Investigaciones Científicas (CSIC), Juan de la Cierva 3, 28006-Madrid, Spain

**Keywords:** alkynes, allenes, gold, heterocyclizations, β-lactams

## Abstract

New gold-catalyzed methods using the β-lactam scaffold have been recently developed for the synthesis of different sized heterocycles. This overview focuses on heterocyclization reactions of allenic and alkynic β-lactams which rely on the activation of the allene and alkyne component. The mechanism as well as the regio- and stereoselectivity of the cyclizations are also discussed.

## Introduction

The chemistry of alkynes and allenes has been extensively studied and many reviews on their preparation and reactivity have been published [1–9]. These compounds show interesting reactivity and selectivity and can lead to complex structures in only a few steps. The last decade has witnessed dramatic growth in the number of reactions catalyzed by gold complexes because of their powerful soft Lewis acidic nature [10–16]. In particular, gold-catalyzed intramolecular addition of oxygen and nitrogen nucleophiles across an allene or a carbon–carbon triple bond is intriguing from the point of view of regioselectivity (*endo* versus *exo* cyclizations) as well as it being one of the most rapid and convenient methods for the preparation of heterocycles. On the other hand, in addition to the key role that β-lactams play in medicinal chemistry, namely, their action against pathogenic bacteria, enzyme inhibition, or gene activation [17–23], the use of 2-azetidinones as chiral building blocks in organic synthesis is now well established [24–28]. Moreover, the cyclic 2-azetidinone skeleton has been extensively used as a template on which to build carbo(hetero)cyclic structures joined to the four-membered ring, using the chirality and functionalization of the β-lactam ring as a stereo-controlling element [29–30]. This overview focuses on gold-catalyzed heterocyclization reactions of allenic and alkynic β-lactams which rely on the activation of the allene and alkyne component. The mechanism as well as the regio- and stereoselectivity of the cyclizations are also discussed.

## Review

### Gold-catalyzed heterocyclizations in allenyl-β-lactams

#### Aminocyclizations

**Scheme 1:**
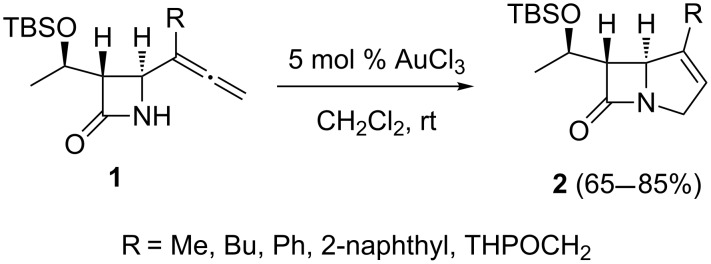
Gold-catalyzed cyclization of 4-allenyl-2-azetidinones for the preparation of bicyclic β-lactams.

The AuCl_3_-catalyzed cyclization of 4-allenyl-2-azetidinones affords bicyclic β-lactams [31]. The former were prepared by the selective introduction of the allenyl group at the C4-position of 2-azetidinones with the help of organo–indium reagents. The best results, among the several reaction conditions examined for the incorporation of the allene moiety in the four-membered ring, were obtained when the organo–indium reagent was generated in situ from the reaction of 2.0 equivalents of indium with 3.0 equivalents of substituted propargyl bromide in the presence of 3.0 equivalents of KI. The best solvent from those that were screened (DMF, THF, C_6_H_6_, and C_6_H_5_CH_3_) was found to be DMF. Because further functionalization of the allene group could potentially lead to the construction of a bicyclic nucleus, an especially intriguing and fundamental problem in the field of carbapenem synthesis, considerable efforts were devoted to the aminocyclization of 4-(1'-methylallenyl)-2-azetidinone derivatives with a variety of catalysts. Although many palladium-based catalysts such as Pd(OAc)_2_, PdCl_2_, [Pd(PPh_3_)_4_], and [Pd_2_(dba)_3_]·CHCl_3_ failed to give the desired cyclized products, exposure of allenyl-β-lactams **1** to 5 mol % AuCl_3_ in CH_2_Cl_2_ produced the bicyclic β-lactam products, i.e., the Δ^1^-carbapenems **2** (Scheme 1). The desired products were produced in good yields for 2-azetidinones with *n*-butyl, THPOCH_2_, phenyl, and 2-naphthyl substituents. It should be mentioned that the cyclization of allenyl-β-lactams **1** is an application of the gold-catalyzed cycloisomerization of α-aminoallenes which was discovered earlier [32–33].

**Scheme 2:**
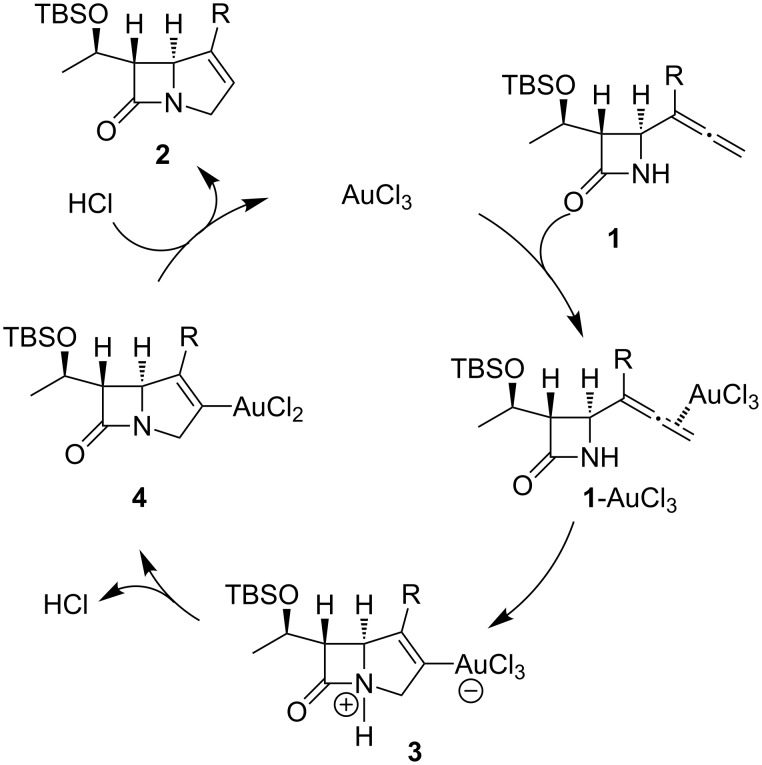
Possible catalytic cycle for the gold-catalyzed cyclization of 4-allenyl-2-azetidinones.

Although the mechanism of the cyclization reaction has not been fully established, a possible reaction pathway has been proposed (Scheme 2) in which AuCl_3_ activates the allene group of 4-allenyl-2-azetidinones **1** to give **1**-AuCl_3_. Subsequent cyclization affords **3**, which then gives a transient vinyl–gold intermediate **4** [34–37]. Protonation of **4** produces bicyclic β-lactams **2** and regenerates AuCl_3_ to continue the catalytic cycle.

#### Oxycyclizations

**Scheme 3:**
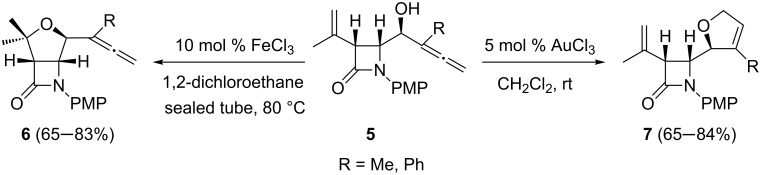
Gold- and iron-catalyzed chemodivergent cyclization of ene-allenols for the preparation of oxacyclic β-lactam derivatives.

Furan, tetrahydrofuran, dihydropyran, and oxepane ether rings are ubiquitous structural units that are extensively encountered in a number of biologically active natural products and functional molecules, and therefore, their stereocontrolled synthesis remains an important research area. On the other hand, the recent resplendent age of gold has been accompanied by the emergence of iron salts as powerful alternatives in view of their inexpensiveness and environmental friendliness [38–40]. The chemodivergent metal-catalyzed heterocyclization of alcohols bearing both an allene and an alkene center has been reported [41]. Starting from 2-azetidinone-tethered ene-allenols **5**, FeCl_3_ was able to catalyze the cyclization chemospecifically in favour of the alkene component to afford exclusively β-lactam–tetrahydrofuran hybrids **6** in good isolated yields (Scheme 3). Besides total chemocontrol, the reaction was regiospecific and only the five-membered ring ether was formed: The isomeric six-membered ring product was not observed. By contrast, when the cyclization of olefinic α-allenols **5** was catalyzed by gold salts (AuCl_3_), allene cycloisomerization adducts **7** were obtained as the sole isomers (Scheme 3). The cyclization of allenyl-β-lactams **5** is an application of the previously reported gold-catalyzed cycloisomerization of α-hydroxyallenes [42–44].

**Scheme 4:**
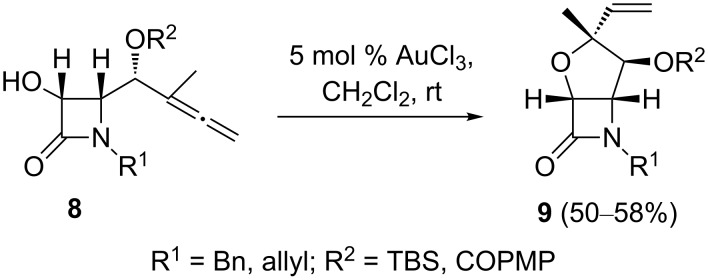
Gold-catalyzed cyclization of hydroxyallenes for the preparation of five-membered oxacyclic β-lactams; COPMP = O=C-C_6_H_4_-OCH_3_.

Similarly to the transition metal-catalyzed reactions of α-allenols which afford heterocyclization products, intramolecular cyclizations of γ-allenols have also attracted a great deal of interest [45–47]. A study of the regioselectivity control during the gold-catalyzed O–C functionalization of 2-azetidinone-tethered γ-allenol derivatives has been published [48–49]. The general reactivity of 2-azetidinone-tethered γ-allenols toward the regioselective hydroalkoxylation reaction was investigated with substrate **8a** (R^1^ = Bn, R^2^ = TBS) using [PtCl_2_(CH_2_=CH_2_)]_2_, AgNO_3_, AuCl and AuCl_3_ as catalysts. [PtCl_2_(CH_2_=CH_2_)]_2_ and AgNO_3_ afforded rather low yields or disappointing diastereomeric mixtures of the bicyclic compound **9a**. Although AgNO_3_ was less diastereoselective than [PtCl_2_(CH_2_=CH_2_)]_2_ (60:40 vs 100:0), it was, nevertheless, a more efficient catalyst and gave adduct **9a** in reasonable yield. Gratifyingly, it was found that Au salts were effective as selective 5-*exo* hydroalkoxylation catalysts. AuCl_3_ was found to be the catalyst of choice because of its superior performance and produced the fused 2-azetidinones **9** in moderate yields (Scheme 4). No regioisomeric products were detected: The reaction gave exclusively the fused five-membered oxacycle.

**Figure 1:**
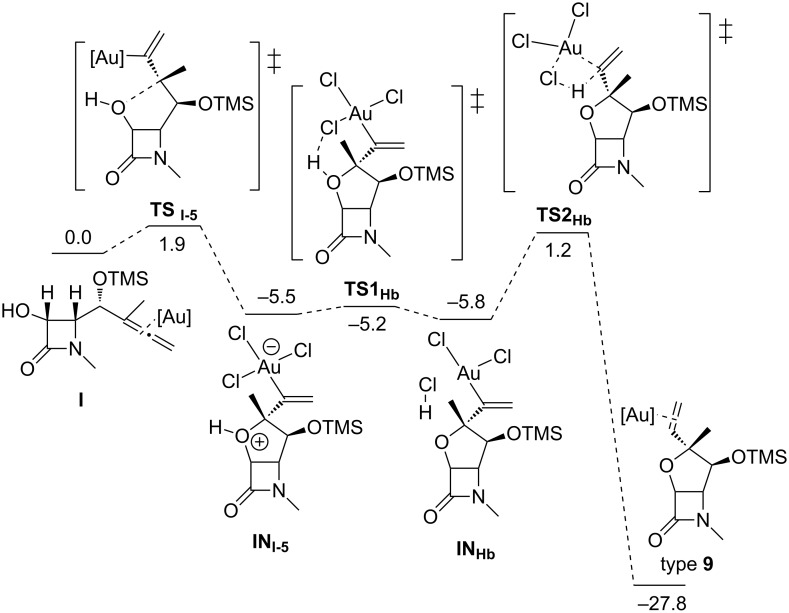
Free energy profile [kcal mol^–1^] for the transformation of γ-allenol **I** into the tetrahydrofuran type **9**.

**Scheme 5:**
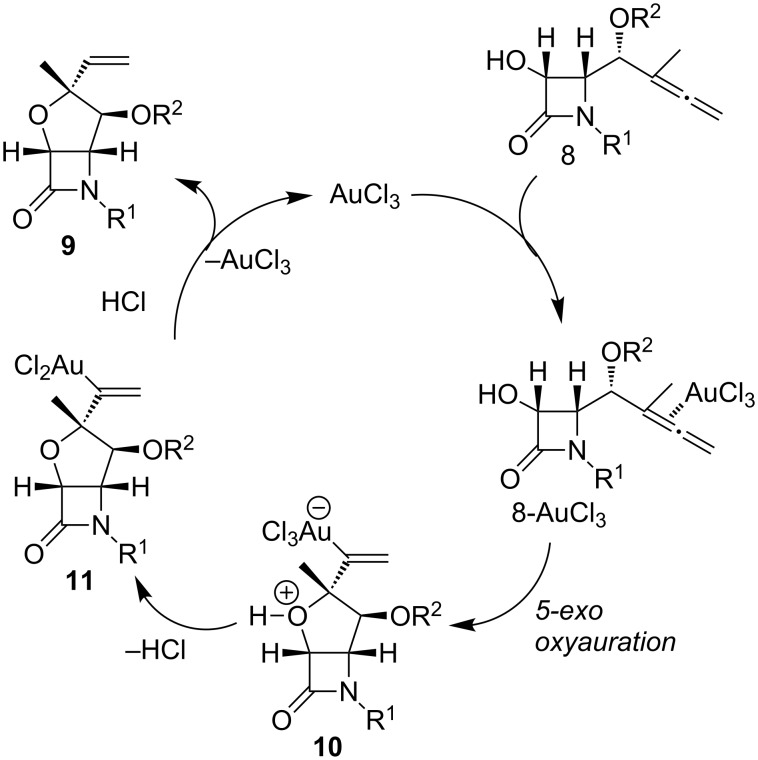
Possible catalytic cycle for the gold-catalyzed cyclization of hydroxyallenes.

A computational study (using density functional theory, DFT) of the above heterocyclization has been carried out [50]. The Au(III)-catalyzed cyclization of γ-allenol **I** (Figure 1) takes place regio- and stereoselectively through a 5-*exo* hydroalkoxylation because of a kinetic preference governed by electronic and steric factors. A possible pathway for the formation of bicyclic compounds **9** from γ-allenols **8** may initially involve the formation of a complex **8**-AuCl_3_ via coordination of the gold trichloride to the proximal allenic double bond which undergoes regioselective 5-*exo* oxyauration to form the zwitterionic species **10**. Loss of HCl followed by protonolysis of the carbon–gold bond of **11** affords products **9** and regenerates the gold catalyst (Scheme 5).

**Scheme 6:**
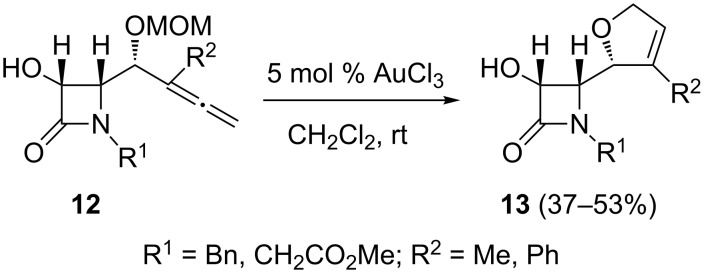
Gold-catalyzed cyclization of MOM-protected α-hydroxyallenes for the preparation of five-membered oxacyclic β-lactams.

**Scheme 7:**
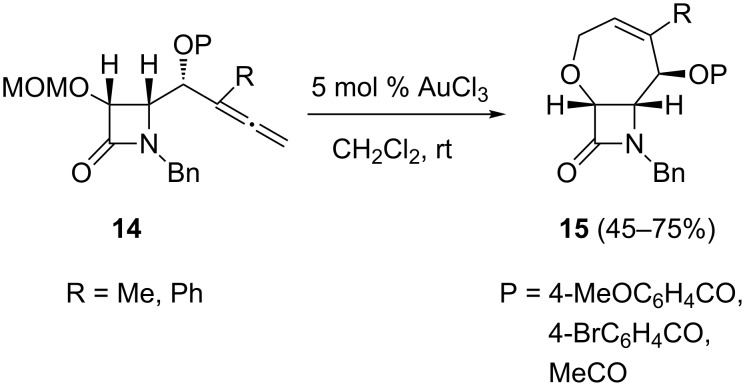
Gold-catalyzed cyclization of MOM-protected γ-hydroxyallenes for the preparation of seven-membered oxacyclic β-lactams.

Having found a solution for the 5-*exo* selective hydroalkoxylation, attention was turned to the more intricate heterocyclization problem associated with the tuning of the regioselectivities of γ-allenol derivatives. It should be mentioned that one of the challenges for modern synthesis is to create distinct types of complex molecules from identical starting materials based solely on catalyst selection. As the stability of the benzoate and TBS-protective groups under the gold-catalyzed conditions had been demonstrated, it was decided to see if (methoxymethyl)oxy substitution has a beneficial impact on the cyclization reactions. In the event, when γ-allenols **12** were treated with AuCl_3_ the 2,5-dihydrofurans **13** were the sole products (Scheme 6). These transformations may involve a chemoselective (5-*endo-trig* versus 7-*endo-trig*) allenol oxycyclization with concomitant MOM ether deprotection. Taking into account the above results, it was decided to see whether the metal-catalyzed preparation of bicycles **9** can be directly accomplished from the MOM protected γ-allenol derivatives **14**. However, when the allenic MOM ethers **14** were treated with AuCl_3_, the 5-*exo* mode was completely suppressed and 7-*endo* cyclization occurred instead to afford bicyclic derivatives **15** in fair yields (Scheme 7). It seems that the reactivity in this type of Au(III)-catalyzed reaction is determined by the presence or absence of a methoxymethyl protecting group at the γ-allenol oxygen atom, thus allenols **8** gave 5-*exo* hydroalkoxylation whilst γ-allenol derivatives **14** exclusively underwent a 7-*endo* oxycyclization. Thus, it has been demonstrated that regioselectivity control in the metal-catalyzed O–C functionalization of γ-allenols can be achieved through the nature of the γ-allenol (free versus protected).

**Scheme 8:**
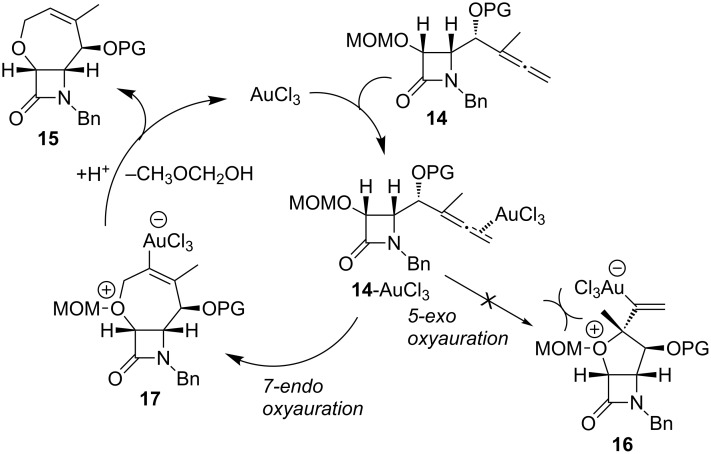
Possible catalytic cycle for the gold-catalyzed cyclization of MOM protected γ-allenol derivatives. PG = Protecting group.

The pathway proposed in Scheme 8 appears valid for the formation of products **15** from MOM protected γ-allenol derivatives **14**. It is presumed that the initially formed allene–gold complex **14**-AuCl_3_ undergoes an intramolecular attack (7-*endo* versus 5-*exo* oxyauration) by the (methoxymethyl)oxy group, giving rise not to species **16** but instead to the tetrahydrooxepine intermediate **17**. Protonolysis of the carbon–gold bond and elimination of methoxymethanol would then liberate the compound **15** with concomitant regeneration of the Au(III) species. Probably, the proton in the last step of the catalytic cycle arises from trace amounts of water present in the solvent or the catalyst. In the presence of the MOM group, 5-*exo* cyclization falters. As calculations reveal, 5-*exo* oxyauration via **16** is restricted by the steric hindrance between the (methoxymethyl)oxy group and the substituents at the quaternary stereocenter.

**Scheme 9:**
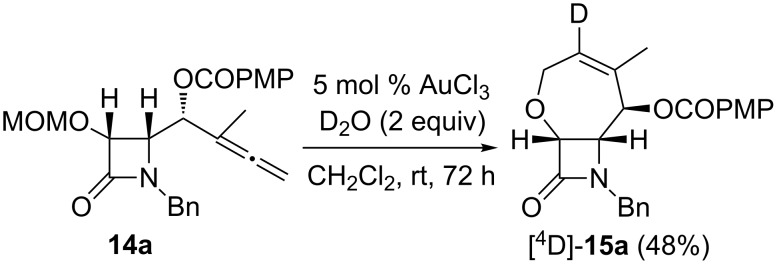
Au(III)-catalyzed heterocyclization reaction of MOM protected γ-allenol derivative **14a**.

With the aim of trapping the organo–gold intermediate to confirm the mechanism of this reaction, deuterium labeling studies with deuterium oxide were performed. Under the same conditions but with the addition of two equivalents of D_2_O, heterocyclization reaction of MOM protected γ-allenol **14a** catalyzed by AuCl_3_ in dichloromethane afforded [^4^D]-**15a** in 48% yield, indicating that a deuterium atom was incorporated at the alkenyl carbon (Scheme 9). In the ^1^H NMR spectrum of [^4^D]-**15a**, the peak for proton H4 at 6.35 ppm was absent which suggests that deuterolysis of the carbon–gold bond in species **17** has occurred. Along with the clarification of the reaction mechanism, it should be pointed out that, although metal-catalyzed oxycyclization reactions of allenes are well-known in hydroxyallenes, the heterocyclization of alkoxyallenes is not an easy task and still remains a significant challenge.

### Gold-catalyzed heterocyclizations in alkynyl-β-lactams

#### Aminocyclizations

**Scheme 10:**
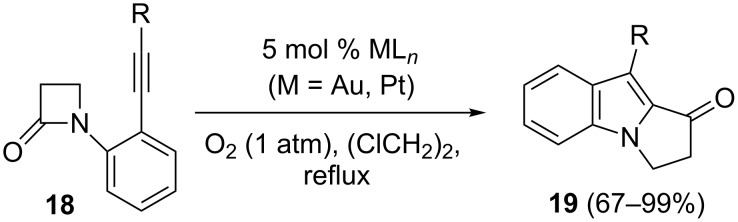
Precious metal-catalyzed formation of benzo-fused pyrrolizinones from *N*-(2-alkynylphenyl)-β-lactams.

The precious metal-catalyzed formation of benzo-fused pyrrolizinones **19** from *N*-(2-alkynylphenyl)-β-lactams **18** has been accomplished (Scheme 10). Platinum was the metal of choice, gold salts being less effective [51]. This cycloisomerization can be viewed as a net intramolecular insertion of one end of the alkyne into the lactam amide bond with concurrent migration of the substituent at the alkyne terminus. An initial *5*-*endo*-*dig* cyclization of the lactam nitrogen to the metal-activated alkyne was proposed, followed by the fragmentation of the lactam amide bond and the formation of an acyl cation.

**Scheme 11:**
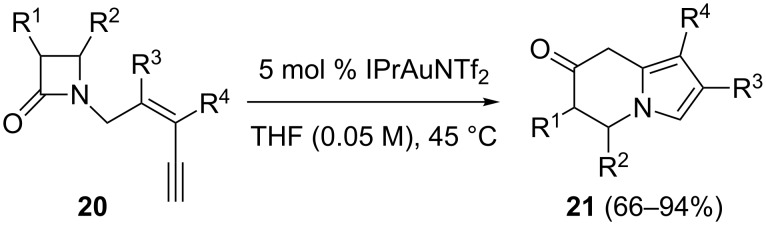
Gold-catalyzed formation of 5,6-dihydro-8*H*-indolizin-7-ones from *N*-(pent-2-en-4-ynyl)-β-lactams.

The above chemistry was extended to non-aromatic substrates, providing a new approach to other *N*-heterocycles [52]. Thus, the benzene ring was substituted by a *cis*-alkene, and a gold-catalyzed synthesis of 5,6-dihydro-8*H*-indolizin-7-ones **21** from *N*-(pent-2-en-4-ynyl)-β-lactams **20** was developed (Scheme 11). Pt(II) and Pt(IV) also catalyzed this reaction, albeit less efficiently. In this reaction, a *5-exo*-*dig* cyclization of the β-lactam nitrogen to the Au-activated C–C triple bond is followed by heterolytic fragmentation of the amide bond to form a reactive acyl cation. While substrates with substituents at the alkyne terminus did not undergo this catalytic reaction, various substituents at the C–C double bond were tolerated, including benzyloxyethyl and cyclohexyl (geminal to the ethynyl group) as well as hexyl and phenyl (vicinal to the lactam), and gave dihydroindolizinones with different substituents at their 1- and 2-positions. Substrates with the C–C double bond embedded in medium-sized rings also reacted well to yield interesting seven- or eight-membered ring fused dihydroindolizinones in good yields. Surprisingly, the corresponding cyclopentene or cyclohexene substrates did not afford the corresponding five- or six-membered ring-fused dihydroindolizinones. After 10 h, the starting materials were mostly unreacted in the case of cyclohexene substrates and partly decomposed in the case of cyclopentene substrates. This method allows an expedient formal synthesis of indolizidine 167B.

#### Oxycyclizations

Transition metal-assisted intramolecular addition of oxygen nucleophiles across a carbon–carbon triple bond is intriguing from the point of view of regioselectivity as well as it being one of the most rapid and convenient methods for the preparation of oxacycles [53–64]. Recently, the gold-catalyzed cycloisomerization and tandem oxycyclization/hydroxylation of 2-azetidinone-tethered alkynols for the synthesis of non-fused, spiro, and fused oxabicyclic β-lactams has been reported [65].

**Scheme 12:**
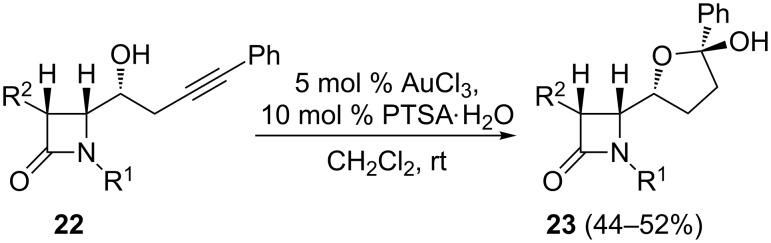
Gold-catalyzed formation of non-fused tetrahydrofuryl-β-lactam hemiacetals from 2-azetidinone-tethered homopropargylic alcohols.

Attempts at a cyclization reaction of terminal alkynols using gold catalysts failed. However, under the appropriate reaction conditions was found that AuCl_3_ could be a good catalyst for the cycloetherification reaction of non-terminal alkynols **22**. Scheme 12 shows that tetrahydrofuryl hemiacetals **23** are accessible as single isomers in fair yields via the gold-catalyzed tandem oxycyclization/hydroxylation reaction of 2-azetidinone-tethered homopropargylic alcohols. In the conversion from alkynols **22** to tetrahydrofuryl hemiacetals **23**, water is required, which is probably provided by trace amounts of water present in the solvent or the catalyst. Additionally, it should be noted that PTSA contains water since the monohydrate is actually employed.

**Scheme 13:**
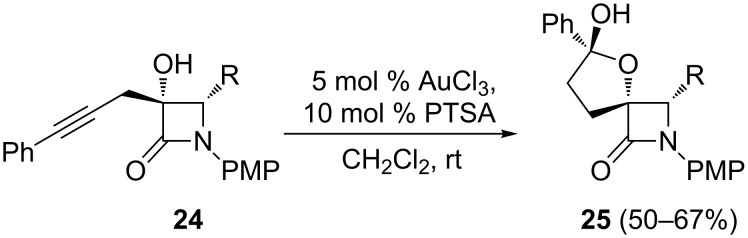
Gold-catalyzed formation of spiro tetrahydrofuryl-β-lactam hemiacetals from 2-azetidinone-tethered homopropargylic alcohols.

**Scheme 14:**
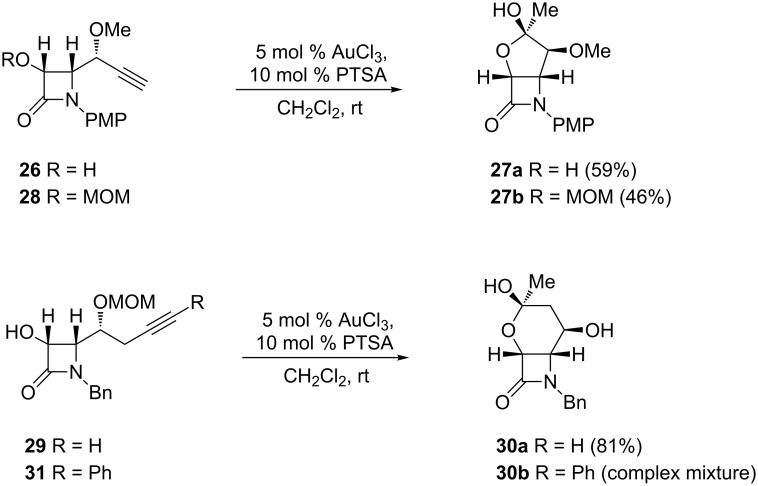
Gold-catalyzed formation of fused tetrahydrofuryl-β-lactam hemiacetals from 2-azetidinone-tethered bis- and tris-homopropargylic alcohols.

In order to determine whether the conclusions drawn from the homopropargylic alcohols **22** could be extrapolated to other alkynols, tertiary carbinols **24** were examined. Under similar gold-catalyzed conditions, spiro β-lactams **25** were obtained as single isomers in good yields (Scheme 13). To further probe the scope of these transformations, gold-catalyzed heterocyclization reactions of alkynols to the fused bicyclic systems was also examined. Indeed, treatment of 2-azetidinone-tethered bishomopropargylic alcohol **26** with AuCl_3_ provided the desired cycloetherification/hydroxylation product **27a** in good yield (Scheme 14). Interestingly, the gold-catalyzed reaction of **28,** with a (methoxymethyl)oxy moiety instead of the free hydroxy group, also proceeded smoothly to give the cyclization product **27b,** albeit in lower yield (Scheme 14). Notably, the observed regioselectivity (5-*exo* cyclization) was unaffected by the presence of a protective group at the hydroxy moiety. These gold-catalyzed oxycyclizations were successfully extended to trishomopropargylic alcohol **29**, which afforded the oxycyclization/hydroxylation adduct **30a** with concomitant MOM cleavage (Scheme 14). In contrast, the presence of a phenyl substituent at the terminal alkyne carbon showed a substantial effect on the reactivity, as illustrated by the fact that phenyl alkynol **31** gave a complex mixture of products.

**Scheme 15:**
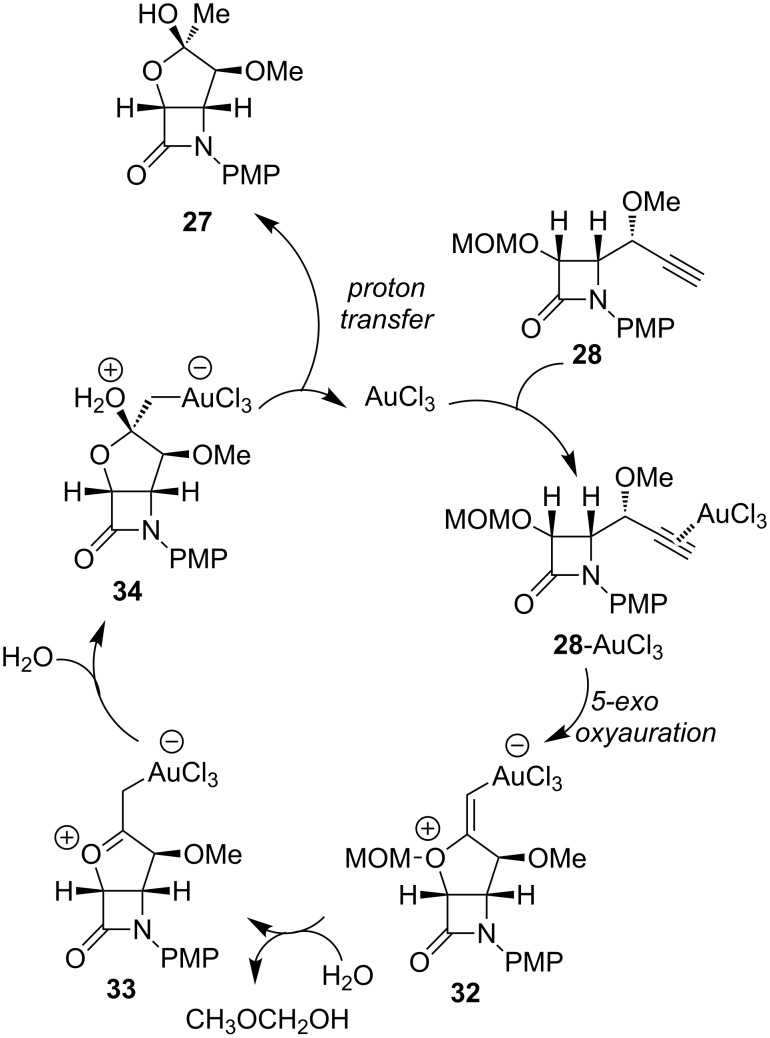
Possible catalytic cycle for the gold-catalyzed cyclization of MOM protected alkynol derivatives.

A conceivable mechanism for the formation of bicyclic tetrahydrofuran **27** from the methoxymethyl ether **28** may initially involve the formation of a π-complex **28**-AuCl_3_ through coordination of the gold trichloride to the alkyne moiety. The initially formed alkyne–gold complex **28**-AuCl_3_ could undergo a regioselective intramolecular attack (5-*exo* versus 6-*endo* oxyauration) by the (methoxymethyl)oxy group to yield the vinyl–gold intermediate **32**. The elimination of methoxymethanol followed by isomerization would lead to the metallaoxocarbenium species **33**. Subsequent nucleophilic attack by water, from trace amounts present in the solvent or the catalyst, from the less hindered face of intermediate **33** would form the ate complex **34**. Deauration and proton transfer leads to adduct **27** with concomitant regeneration of the Au(III) species (Scheme 15).

**Scheme 16:**
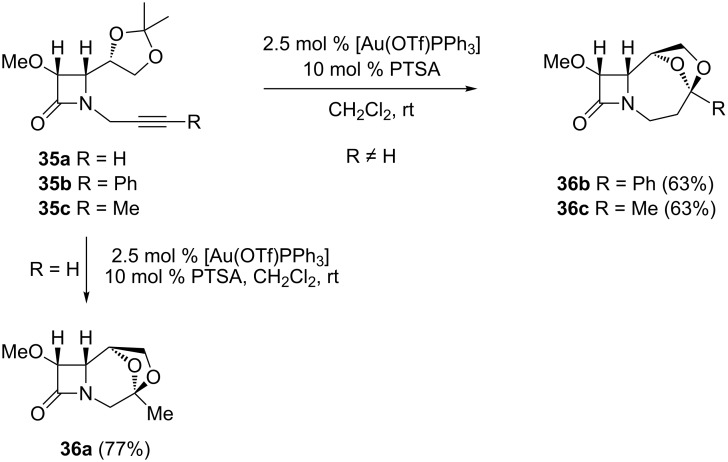
Gold/Brønsted acid co-catalyzed formation of bridged β-lactam acetals from 2-azetidinone-tethered alkynyl dioxolanes.

Regiocontrolled gold/Brønsted acid co-catalyzed direct bis-heterocyclization of alkynyl-β-lactams allows the efficient synthesis of optically pure tricyclic bridged acetals bearing a 2-azetidinone nucleus [66–67]. Treatment of the terminal alkyne **35a** with the catalytic system AuCl_3_/PTSA gave the desired ketal **36a**. Appreciable amounts of a polar ketone arising from alkyne hydration were also produced. Fortunately, the [AuClPPh_3_]/AgOTf/PTSA system demonstrated better activity. Interestingly, in contrast to the precious metal/acid co-catalyzed reaction of terminal alkynyl dioxolane **35a**, which leads to the 6,8-dioxabicyclo[3.2.1]octane derivative **36a** (proximal adduct), the reaction of alkynyl dioxolanes **35b** and **35c**, substituted at the terminal end gave under identical conditions the 7,9-dioxabicyclo[4.2.1]nonane derivatives **36b** and **36c** (distal adducts) as the sole products (Scheme 16), through an exclusive 7-*endo*/5-*exo* bis-oxycyclization by initial attack of the oxygen atom on the external alkyne carbon. Competition between the initial 6-*exo* and 7-*endo* oxycyclizations appears to favor the latter, despite that fact that a priori this should be energetically more demanding.

## Conclusion

In summary, regiocontrolled gold-catalyzed heterocyclization reactions of 2-azetidinone-tethered allenes and alkynes which lead to a variety of oxa- and azacycles have been developed. Density functional theory (DFT) calculations were performed to obtain insight on various aspects of this reactivity and indicated the selective activation of the allene and alkyne component.
